# Global Reach of an Online COVID-19 Course in Multiple Languages on OpenWHO in the First Quarter of 2020: Analysis of Platform Use Data

**DOI:** 10.2196/19076

**Published:** 2020-04-27

**Authors:** Heini Utunen, Ngouille Ndiaye, Corentin Piroux, Richelle George, Melissa Attias, Gaya Gamhewage

**Affiliations:** 1 Learning and Capacity Development Unit World Health Organization Health Emergencies Programme World Health Organization Geneva Switzerland

**Keywords:** online learning, OpenWHO, novel coronavirus, COVID-19, coronavirus, pandemic, WHO, e-learning, MOOC, public health

## Abstract

**Background:**

At the onset of the coronavirus outbreak, the World Health Organization’s (WHO) Health Emergencies Learning and Capacity Development Unit, together with the WHO’s health technical lead on coronaviruses, developed a massive open online course within 3 weeks as part of the global response to the emergency. The introductory coronavirus disease (COVID‑19) course was launched on January 26, 2020, on the health emergencies learning platform OpenWHO.org.

**Objective:**

The aim of this paper is to investigate the geographic reach of different language courses accessed by a worldwide audience seeking information on COVID-19. Users’ professional identities and backgrounds were explored to inform course owners on the use case. The course was developed and delivered via the open-access learning platform OpenWHO.org. The self-paced resources are available in a total of 13 languages and were produced between January 26 and March 25, 2020.

**Methods:**

Data were collected from the online courses’ statistical data and metrics reporting system on the OpenWHO platform. User patterns and locations were analyzed based on Google Analytics and the platform’s own statistics capabilities, and data sets were overlaid. This analysis was conducted based on user location, with the data disaggregated according to the six WHO regions, the top 10 countries, and the proportion of use for each language version. Data included affiliation, gender, age, and other parameters for 32.43% (52,214/161,007) of the users who indicated their background.

**Results:**

As of March 25, 2020, the introductory COVID-19 course totaled 232,890 enrollments across all languages. The Spanish language course was comprised of more than half (n=118,754, 50.99%) of all course enrollments, and the English language course was comprised of 38.21% (n=88,988) of enrollments. The WHO’s Region of the Americas accounted for most of the course enrollments, with more than 72.47% (138,503/191,130) enrollment across all languages. Other regions were more evenly distributed with less than 10% enrollment for each. A total of 32.43% (52,214/161,007) of users specified a professional affiliation by choosing from the 12 most common backgrounds in the OpenWHO user profiles. Before the COVID-19 pandemic, users were spread over the 11 distinct affiliations, with a small fraction of users identifying themselves as “Other.” With the COVID-19 introductory course, the largest number of users selected “Other” (16,527/52,214, 31.65%), suggesting a large number of users who were not health professionals or academics. The top 10 countries with the most users across all languages were Argentina, Chile, Colombia, Ecuador, India, Mexico, Peru, Spain, the United Kingdom, and the United States.

**Conclusions:**

The online course has addressed a worldwide learning need by providing WHO’s technical guidance packaged in simple formats for access and use. The learning material development was expedited to meet the onset of the epidemic. Initial data suggest that the various language versions of the course, in particular Spanish, have reached new user groups, fulfilling the platform’s aim of providing learning everywhere to anyone that is interested. User surveys will be carried out to measure the real impact.

## Introduction

### Background

The focus of this study is the World Health Organization’s (WHO) health emergencies platform OpenWHO.org, which hosts online learning resources for outbreaks and epidemics. OpenWHO is an open source online platform adjusted for low bandwidths with mobile and download capabilities. This free, web‑based knowledge transfer platform was designed for massive, real time use during a pandemic and has been in a real test during the early part of 2020 in offering free online courses to improve the response and preparedness for coronavirus disease (COVID-19).

The COVID-19 resources are hosted on two learning channels on the platform: one for courses in official UN languages and a second for courses in additional national languages. The first course related to COVID-19, “Introduction to emerging respiratory viruses, including COVID-19: methods for detection, prevention, response and control,” was launched on OpenWHO on January 26, 2020, following the first WHO Emergency Committee meeting on January 22 and 23, 2020.

The course has four modules:

1. Introduction to emerging respiratory viruses, including COVID-19
2. Detecting emerging respiratory viruses, including COVID-19: surveillance and laboratory investigation
3. Risk communication and community engagement
4. Preventing and responding to an emerging respiratory virus, including COVID‑19

This first edition of the course includes introductory information on the novel coronavirus and other coronaviruses, and basic information for anyone wanting to understand the new epidemic. By the end of the course, learners should be able to describe the nature of emerging respiratory viruses, how to detect and assess an outbreak, and strategies for preventing and controlling outbreaks due to novel respiratory viruses, as well as what strategies should be used to communicate risk and engage communities. The course was packaged and presented through PowerPoint slide decks, and video recordings by WHO health experts in different areas of work were added during the following days and weeks. This simple packaging allows for material to be used on multiple devices, in low-bandwidth settings, and through an offline function in the mobile app. The packaging of the online courses with videos and slide decks also supports the ability to update the material to reflect the frequently changing and updated WHO technical guidance.

Due to the changing content during the first months of the Public Health Emergency of International Concern and the first weeks of the pandemic, the OpenWHO team did not make available any quizzes as is usual for other courses on the platform and, thus, was not providing a certificate of completion. These features will be added once the technical content can be considered more final and established.

**Figure 1:**
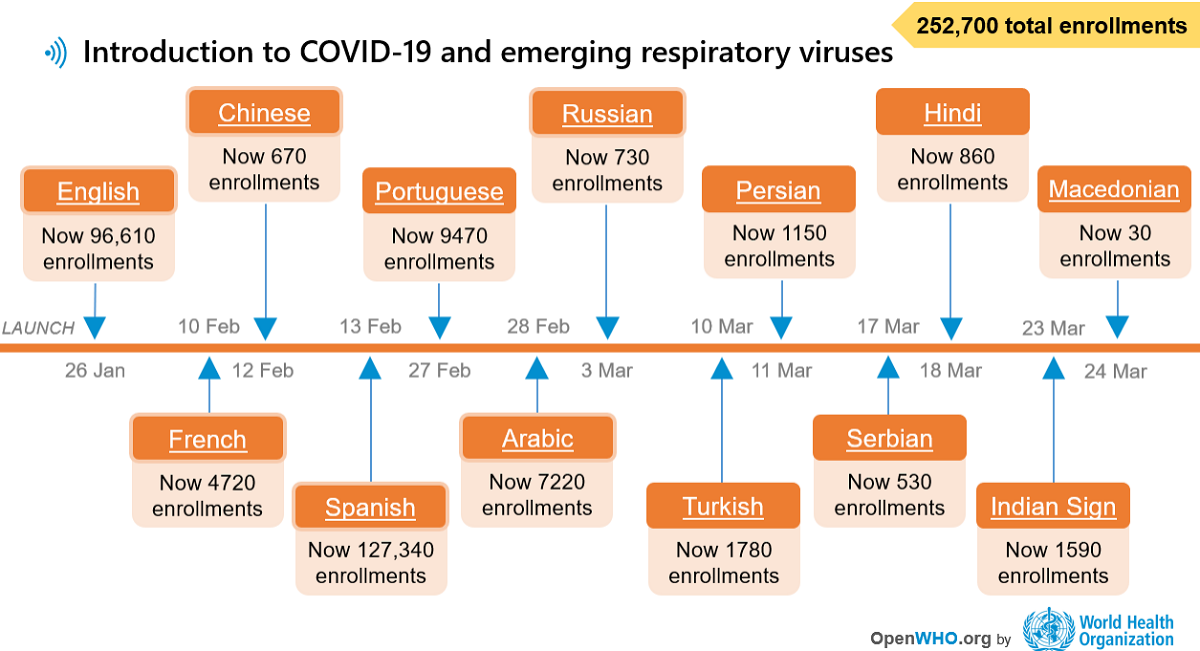
Introduction to coronavirus disease course languages and enrollment figures as of March 27, 2020.

The timeline in Figure 1 shows the launch of the course in different languages and the total enrollments as of March 27, 2020, the day the OpenWHO platform reached 1 million enrollments.

The course was published on January 26, 2020, in English and was gradually published in all other UN languages. Another 7 national languages were produced by the courtesy of dozens of volunteers providing spontaneous translation offers from WHO country and regional offices and headquarters, as well as volunteers from public health institutes and educational units. All volunteers were seeking to provide support to maximize the local-level uptake of courses for an effective response to the pandemic. Among other efforts, OpenWHO released an introductory video to COVID-19 in Indian sign language, the first sign language resource on the platform.

**Figure 2:**
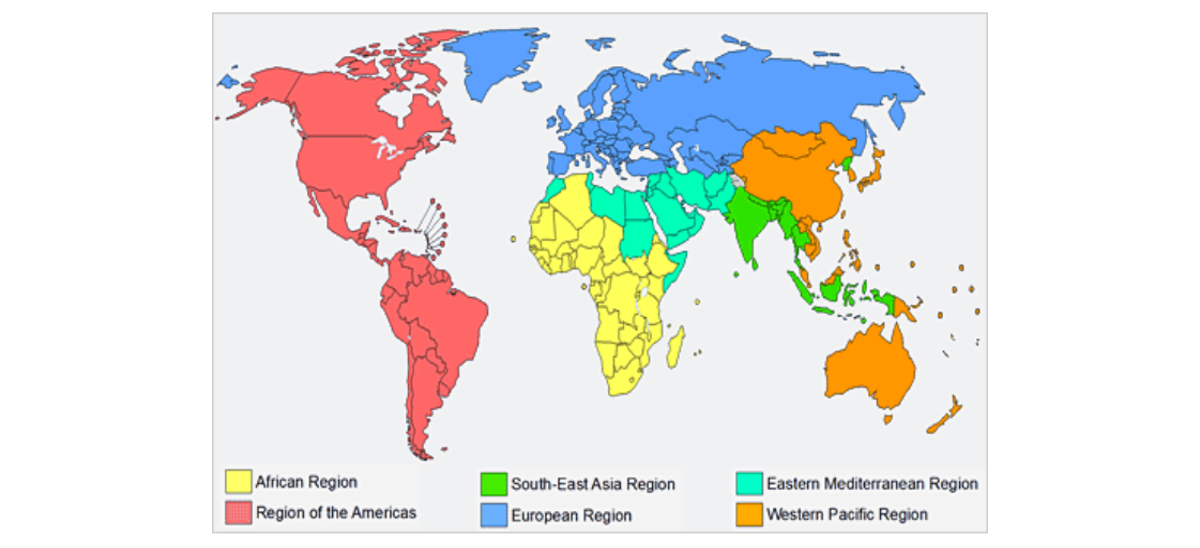
World Health Organization regions.

This study looks at the course use in all of the WHO regions. The WHO Member States are grouped into six WHO regions: African Region, Region of the Americas, Eastern Mediterranean Region, European Region, South-East Asia Region, and Western Pacific Region, as shown in Figure 2.

### Relevant Literature

According to the WHO [1], “health literacy represents the cognitive and social skills that determine the motivation and ability of individuals to gain access to understand and use information in ways which promote and maintain good health.” Research has found that the WHO is a respected source of accurate health information during epidemics, suggesting that the organization has a platform to shape health behavior, which less‑trusted sources such as governments may lack [2]. The OpenWHO team has focused on designing knowledge transfer resources for health emergencies in formats and languages that are suitable for frontline responders and affected communities. This approach has prioritized multi-language production, recognizing that language can be a key obstacle to health literacy [3].

A 2015 study in Kenya by Translators Without Borders found that providing health information in Swahili—the lingua franca throughout the country—produced a significant increase in comprehension compared with providing the same information in English [4]. A 2019 study by the same organization, which partners with the WHO team on many translations, found that the local form of Swahili was the most effective language for risk communication and community engagement for the Ebola response in Goma, Democratic Republic of the Congo, compared with the French and standard Swahili languages [5]. Providing health information in individuals’ native languages has also shown to improve knowledge about illnesses and medications in a patient population in Sri Lanka, as well as the understanding of oral health information among Vietnamese-speaking mothers in Australia [6,7].

Materials produced for OpenWHO are designed with additional accessibility considerations in mind. The resources are offered as downloadable slides that combine images and short texts, which can be read on a mobile device. Video and audio formats are also being integrated for those with strong oral cultures. In addition, the open-access nature of the platform can empower individuals who are more health literate to strengthen the health literacy of their communities, particularly in group-oriented societies. In a cholera-endemic neighborhood in Ghana, researchers found that household units impacted individual health literacy; nearly three‑quarters of households surveyed followed suggestions from household members on how to prevent cholera [8].

By making materials from a reputable source available in multiple languages and in easily accessible, portable formats, OpenWHO's COVID-19 resources aim to contribute to improved health literacy.

## Methods

This article investigates the geographic reach of different language courses accessed by the worldwide audience population while seeking health information on COVID-19. The users’ professional identities are explored to inform the course owners on the use case. The course was developed and delivered via the learning platform OpenWHO.org. The self-paced introductory course was provided in a total of 13 languages between January 26 and March 25, 2020. The 13 languages were Arabic, Chinese, English, French, Hindi, Indian sign language, Macedonian, Persian, Portuguese, Russian, Serbian, Spanish, and Turkish.

This study’s preliminary objective was to demonstrate the rapid surge of learners accessing the digitized learning materials as the COVID-19 epidemic grew into a pandemic during the early part of 2020. The aim of this study was to obtain a better understanding of the origin and type of people who sought access to online learning related to the emerging health crisis.

Statistical data for the identical courses in 13 languages were generated. More in-depth analysis was carried out on the English and Spanish language courses given the large use case in these two languages—89.20% (207,742/232,890) of all learners.

The data was collected from the online courses’ statistical data and metrics reporting system on the OpenWHO platform. User patterns and locations were analyzed based on Google Analytics and the OpenWHO platform’s own statistical capabilities, and data sets were overlaid.

This snapshot analysis was conducted based on user location with the data disaggregated according to the six WHO regions, the top 10 countries with the most users, and the proportion of use for each language version. Data included affiliation, gender, primary language, age, and other parameters for approximately 30% of users who indicated their background.

## Results

### Introductory COVID-19 Course User Metrics

**Figure 3:**
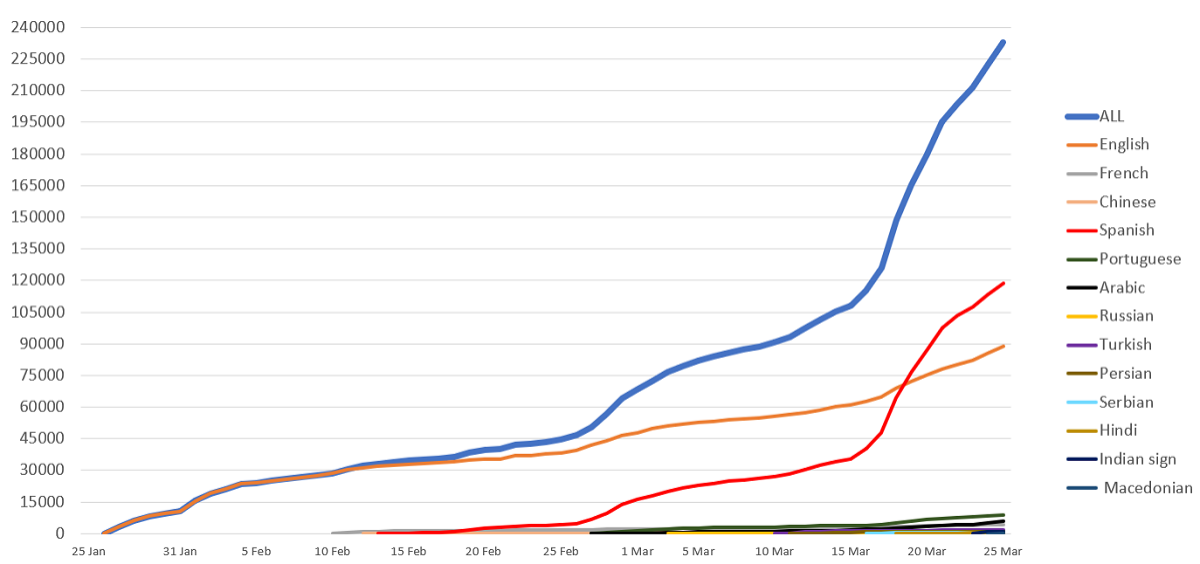
Introduction to coronavirus disease course use by language as of March 25, 2020.

During the first 2 months of the course’s availability (January 26 to March 25, 2020), all 13 languages combined gathered 232,890 enrollments. The use of materials intensified after the declaration of the COVID-19 pandemic on March 11, 2020 (Figure 3).

The two most popular languages were the Spanish language course, which comprised half (n118,754/232,890, 50.99%) of all introductory COVID-19 course enrollments as of March 25, 2020, and the English language course, which comprised 38.21% (n=88,988) of enrollments. Despite being launched 2 weeks later, the Spanish version rapidly surpassed the original English course on March 19, 2020, becoming the main language driving the increase in enrollment. The English and Spanish course users jointly amounted to 210,000 users, representing 90.17% of all users across all language versions.

**Figure 4:**
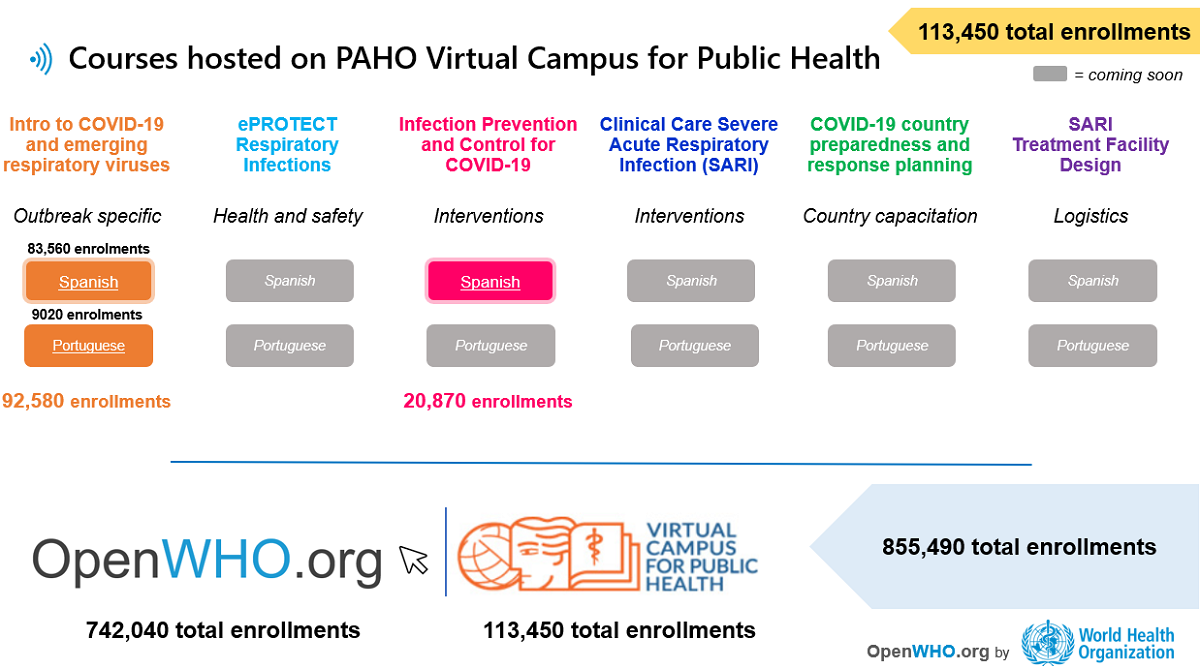
OpenWHO courses hosted on the Pan-American Health Organization virtual campus as of March 27, 2020. COVID-19: coronavirus disease.

As expected, the number of accumulated enrollments across all language versions of the course rose steadily with each new language version launched (Figure 4).

In terms of user locations, the top 10 countries with the most users across all languages, accounting for (40,893/57,763) 70.79% of the total course users, were Ecuador (n=9521), Mexico (n=8236), Colombia (n=6137), Chile (n=4067), the United States (n=3836), Argentina (n=3609), Spain (n=1766), the United Kingdom (n=1499), India (n=1117), and Peru (n=925).

This makeup represents a marked shift in comparison with other top courses on the platform. For example, prior to the current outbreak of COVID-19, the platform’s most popular emergency-related course, eProtect Ebola (offered in English and French), consisted primarily of users from Africa, Europe, and North America, with no South or Central American countries appearing in the top 20 for either language version of the course. This pattern reflected what was a general trend across the platform in the months preceding the launch of the introductory COVID-19 course; the platform’s top 5 countries most commonly consisted of, in descending order, the United States, India, the United Kingdom, Portugal, and Nigeria, with only the presence of India breaking the aforementioned trend. As such, the launch of the introductory COVID-19 course has brought with it a change in the demographic of users on the platform, with a marked increase in traction from Central and South America.

### Geographical Distribution of Users

**Figure 5:**
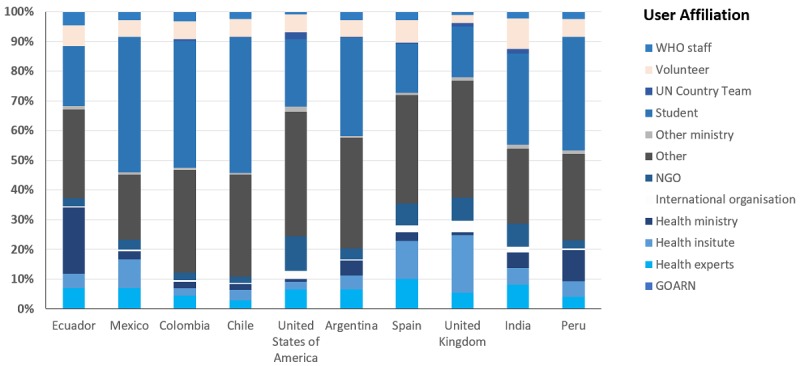
Users from the top 10 countries and their affiliations. GOARN: Global Outbreak Alert and Response Network; NGO: nongovernmental organization; WHO: World Health Organization.

The worldwide distribution of users is displayed in Figure 5 according to the WHO regions. The Region of the Americas accounted for most of the enrollments in the course, with more than (138,503/191,130) 72.47% of the total enrollments across all languages. Other regions were more evenly distributed: African Region (n=7643, 4.00%), Eastern Mediterranean Region (n=12,945, 6.77%), European Region (n=18,259, 9.55%), South-East Asia Region (n=7245, 3.79%), Western Pacific Region (n=5291, 2.77%), and territories (n=1244, 0.65%).

Users originating from the Region of the Americas accounted for over half of the enrollments in the Spanish language course. The second most popular language choice for users in the region was English, with a majority of English-course users enrolling from the United States of America, totaling more than a quarter (n=23,794/138,503, 28.75%) of the total English course users (Table 1).

Each language version provided interesting findings. The Serbian language course, for example, was used more in 4 other countries (Ecuador, Chile, Colombia, and Bosnia and Herzegovina) than it was in Serbia. The Indian sign language course attracted 1000 enrollments in the first 24 hours, with the largest use in the city of Baghdad, Iraq. Just after Portugal, the second top country for the Portuguese course was Mexico. For this version, almost as many enrollments came from Mexico City as from Lisbon. After Lisbon and Mexico City, 3 other Spanish-speaking cities were in the top 5 for the Portuguese version (Bogotá and Medellín in Colombia, and Quito in Ecuador), exceeding the numbers of enrollments from any other Portuguese-speaking city. São Paulo and Rio de Janeiro (Brazil) were the sixth and seventh top cities, respectively, for the Portuguese version of the course. For the French course, the third top city was not French-speaking as one would expect; Mexico City came right after Paris, France, and Bukavu, Democratic Republic of the Congo.

The introductory course was the most popular in the Region of the Americas, with almost three-fourths of the total enrollments across all languages concentrated in this region. This trend might be explained in part by the language availability of the course for the first month, which was limited to English and Spanish along with the French and Chinese versions (Figure 1). Totaling 54.00% (103,207/191,130) of the total course enrollments, the Spanish version had 95.53% (98,554/103,207) of its enrollments from the Region of the Americas. With 37.03% (70,774/191,130) of the total course enrollments, the English version had 46.39% (32,841/70,774) of its enrollments from this region. The latter statistic speaks to the more even distribution of the 70,744 enrollments in the English language course across the other WHO regions: African Region 8.44% (n=5975), Eastern Mediterranean Region 12.15% (n=8597), European Region 15.82% (n=11,194), South-East Asia Region 9.24% (n=6539), Western Pacific Region 6.96% (n=4924), and the territories 0.99% (n=704). Even taking into consideration the gradual release of the language versions’ impact on these statistics, it is worth noting that no other languages had similar slopes as those seen for the Spanish and English versions (Figure 3).

The European Region came second with 9.55% (18,259/191,130) of the total enrollments, mainly distributed between the English (11,194/18,259, 61.31%) and Spanish (n=3942, 21.59%) versions of the course. The Eastern Mediterranean Region comprised 6.77% (12,945/191,130) of the total enrollments, mainly distributed between the English (n=8597/12,945, 66.41%) and Arabic (n=3419, 26.41%) versions. These proportions might also be explained by the release of the Arabic version 1 month after the English version. The African and South-East Asia regions each made up about 4% of the total enrollments, with the main language versions used being English (5975/7643, 78.18%) and French (n=1255, 16.42%) for the African Region and primarily English (6539/7245, 90.25%) for the South-East Asia Region. This was similar to the Western Pacific Region, which comprised of 2.77% (5291/191,130) of the total course enrollments. The English course was accessed by 93.06% (4924/5291) of the enrollments in this region. The territories comprised 0.65% (1244/191,130) of the total course enrollments, also mainly using the English (704/1244, 56.59%) and Spanish (n=341, 27.41%) versions.

**Table 1:** Introductory course use overview by World Health Organization region and language used for course enrollments (N=191,130).

Region	Total, n (%)	English, n (%)	French, n (%)	Russian, n (%)	Hindi, n (%)	Spanish, n (%)	Persian, n (%)	Portuguese, n (%)	Serbian, n (%)	Turkish, n (%)	Arabic, n (%)	Chinese, n (%)
All regions	191,130 (100.00)	70,774 (37.03)	3398 (1.78)	486 (0.25)	348 (0.18)	103,207 (54.00)	716 (0.37)	5934 (3.10)	296 (0.15)	1274 (0.67)	4208 (2.20)	489 (0.26)
AFRO^a^	7643 (4.00)	5975 (3.13)	1255 (0.66)	3 (0.00)	3 (0.00)	97 (0.05)	36 (0.02)	123 (0.06)	6 (0.00)	63 (0.03)	68 (0.04)	14 (0.01)
AMRO^b^	138,503 (72.47)	32,841 (17.18)	915 (0.48)	98 (0.05)	10 (0.01)	98,554 (51.56)	193 (0.10)	4899 (2.56)	193 (0.10)	462 (0.24)	188 (0.10)	150 (0.08)
EMRO^c^	12,945 (6.77)	8597 (4.50)	284 (0.15)	15 (0.01)	10 (0.01)	137 (0.07)	264 (0.14)	56 (0.03)	21 (0.01)	112 (0.06)	3419 (1.79)	30 (0.02)
EURO^d^	18,259 (9.55)	11,194 (5.86)	821 (0.43)	354 (0.19)	5 (0.00)	3942 (2.06)	156 (0.08)	806 (0.42)	68 (0.04)	456 (0.24)	395 (0.21)	62 (0.03)
SEARO^e^	7245 (3.79)	6539 (3.42)	71 (0.04)	11 (0.01)	313 (0.16)	72 (0.04)	39 (0.02)	28 (0.01)	7 (0.00)	128 (0.07)	16 (0.01)	21 (0.01)
WPRO^f^	5291 (2.77)	4924 (2.58)	31 (0.02)	5 (0.00)	7 (0.00)	64 (0.03)	22 (0.01)	20 (0.01)	1 (0.00)	48 (0.03)	19 (0.01)	150 (0.08)
Territories	1244 (0.65)	704 (0.37)	21 (0.01)	0 (0.00)	0 (0.00)	341 (0.18)	6 (0.00)	2 (0.00)	0 (0.00)	5 (0.00)	103 (0.05)	62 (0.03)

^a^AFRO: African Region.

^b^AMRO: Region of the Americas.

^c^EMRO: Eastern Mediterranean Region.

^d^EURO: European Region.

^e^SEARO: South-East Asia Region.

^f^WPRO: Western Pacific Region.

### Spanish Course Use Case

The highest Spanish course use was in Ecuador (n=36,345), Mexico (n=26,141), Colombia (n=19,733), Chile (n=11,793), and Argentina (n=11,711). The highest English course use was in the United States (n=12,250), Mexico (n=7659), Ecuador (n=5805), India (n=5296), and the United Kingdom (n=4052), with Colombia and Argentina also making it to the top 10.

Of the total users who indicated their language of preference, 61.45% (60,800/98,937) selected Spanish. This language preference helps the OpenWHO team further target the courses and prioritize languages. The other preferred languages selected were English (n=31,837, 32.18%), French (n=2369, 2.39%), Portuguese (n=1374, 2.17%), Arabic (n=1374, 1.39%), Russian (n=249, 0.25%), and Chinese (n=160, 0.16%).

The indicated language preference correlated with the language course use; however, as the option for the preferred language only included the six UN official languages and Portuguese, the OpenWHO team was not able to capture if there were any national or local languages popular in addition to the official UN languages.

The same introductory course was published on the Pan-American Health Organization’s (PAHO) virtual campus (VC) in Spanish on February 11 and in Portuguese on February 28, 2020. This paper and the data exclude the 92,000 users of these identical courses on the PAHO VC, which would bring the merged worldwide user numbers even higher (Figure 4).

### User Background Information

Before the COVID-19 pandemic, users were spread over the 11 distinct affiliation options provided by the platform, with a small fraction of users identifying themselves as “Other.” With the epidemic accelerating into a pandemic, the largest number of the COVID-19 introductory course users selected “Other,” suggesting a large number of users who were not health professionals or academics. Students were the largest identifiable group among those who indicated their affiliation. Health ministries and health experts made up 14.21% (7417/52,214), and UN country teams and WHO staff amounted to 4.00% (n=2087; Table 2).

**Table 2:** Users’ professional affiliations for the total users who specified their professional affiliation (n=52,214).

Affiliations	Users, n (%)
Other	16,527 (31.65)
Student	14,945 (28.62)
Health ministry	4243 (8.13)
Volunteer	3330 (6.38)
Health experts	3174 (6.08)
Health institute	3141 (6.02)
Nongovernmental organization	2981 (5.71)
World Health Organization staff	1525 (2.92)
International organization	995 (1.91)
Other ministry	730 (1.40)
UN country team	562 (1.08)
Global Outbreak Alert and Response Network	61 (0.12)

When looking at the top countries and user affiliations in Figure 5, students accounted for a large proportion of the users coming from those countries. For Chile, Colombia, and Mexico, for instance, students represented 45.66% (1857/4067), 42.85% (2707/6317), and 45.70% (3764/8236), respectively, of the total enrollments. As mentioned earlier for the overall analysis, Figure 5 shows that many users were not affiliated with a specific health background. If we examine the figure, we find that the percentage of users who selected “Other” as their affiliation varied from 21.90% (1803/8236) in Mexico to 39.22% (588/1499) and 41.82% (1604/3836) in the United Kingdom and the United States, respectively. Health institutes were also represented in the affiliations for the users in the top 10 countries. The share of health institute workers was the highest for the United Kingdom at 19.48% (292/1499). Another interesting finding was the attraction of health ministry representatives, especially significant for Ecuador, as they represented 22.27% (2120/9521) of the total users for the country. Besides Ecuador, Peru (96/925, 10.37%) is the only other country reporting enrollments from health ministry professionals that reached at least 10%. Other health experts also enrolled in the course; they represented, for instance, 9.85% (174/1766) of the total enrollments reported from Spain. Volunteers were also represented in all of the top 10 countries, with India reporting the highest percentage of this specific user affiliation in 10.29% (115/1117) of its enrollments.

The course registrations also suggested that women (33,216/57,712, 57.55%) were a larger user group than men (n=24,383, 42.25%). In addition, 0.20% (113) of users identified as “Other”.

**Figure 6:**
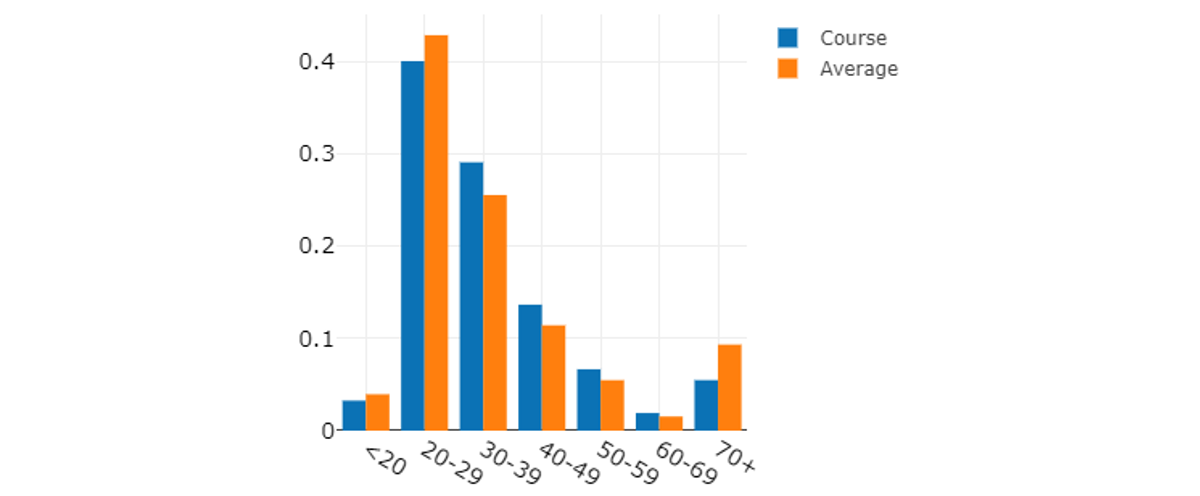
English course use by age groups.

**Figure 7:**
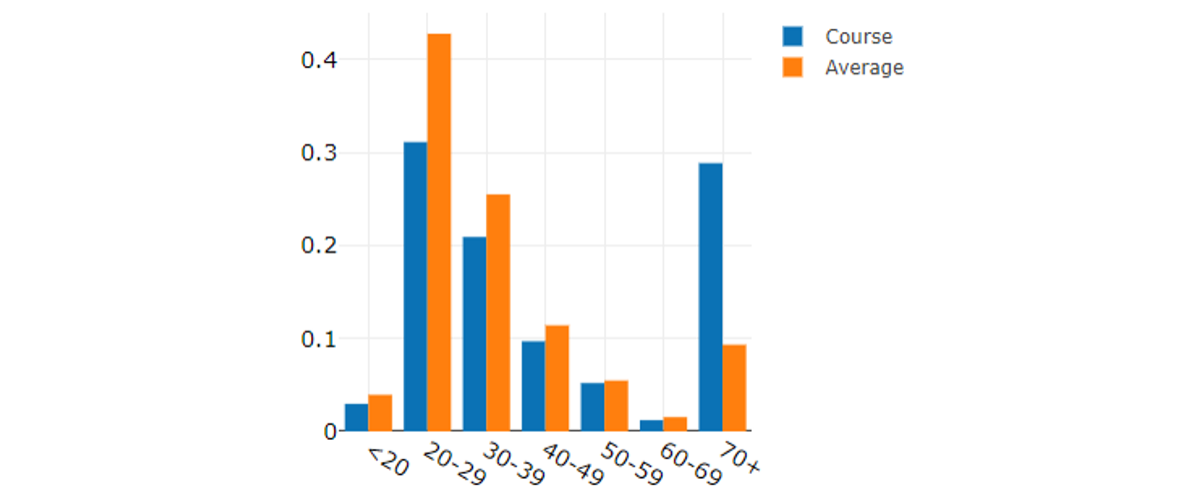
Spanish course use by age groups.

When comparing the English and Spanish course age groups (Figures 6 and 7), the Spanish course had a large cohort of participants who were 70 years or older, which was much higher than the platform average or the English course. As COVID-19 was stated to be impacting older people the most, this use case comes as no surprise. On average, the English course had a younger use case than the platform average, especially in the age groups 30-39 years and 40-49 years.

### Spanish and English Courses and Completion Rates

A total of 36.43% (14,382/39,475) of participants that enrolled in the Spanish introductory course completed all six learning items within the course. In comparison with industry standards, which place the completion rate for massive open online courses (MOOCs) at 7.40% (3700 completions per 50,000 enrollments) [9], this rate is high. Again, in relation to the Spanish introductory course, 44.66% (17,630/39,475) of the participants completed at least 80.00% of the course material, and 49.09% (n=19,377) visited at least 60.00% of the course material. When the completion rate was calculated across all language versions of the course, this trend continued, with 21.63% (16,003/73,980) of users visiting 100.00% of the course items, 27.62% (n=20,433) visiting at least 80.00%, and 32.54% (n=24,075) of users visiting at least 60.00% of the total learning resources in the course.

Participants enrolled in the English version did not perform as well. Only 2.94% (813/27,639) of the participants who enrolled in the English course completed all course material, and only 6.61% (n=1828) completed at least 80.00% of the course material, and 16.65% (n=4603) visited at least half of all course items. Further investigation is required to determine the cause of the discrepancy between the completion rates for the English version of the course compared with the completion rate across all language versions combined. Unlike the subsequent language versions, the English introductory COVID-19 course was assembled over a period of several weeks, with new materials being made available as they were constructed and cleared by the technical experts responsible. In contrast, most of the subsequent language versions were launched as full packages with all course items available at once. This difference could begin to explain the discrepancy in completion rates, as the first set of users who enrolled in the English course would have had to return to the course at later dates to view new material as it was added.

### Platform User Surge During the Early Weeks of the COVID-19 Pandemic

Since the launch of the first course related to COVID-19 on OpenWHO on January 26, 2020, the number of unique learners on the platform increased seven times, from 90,700 unique learners to 629,500 as of March 25. The introductory course brought the largest number of new learners along with the Infection Prevention and Control for COVID-19 course.

In the 2.5 years of operations prior to the coronavirus pandemic of 2020, outbreak-related learning resources were each used by thousands of users, with some courses such as eProtect occupational health and safety for Ebola and Antimicrobial Stewardship reaching up to 20,000-30,000 users over 2 years of the course’s life span. The two most popular COVID-19 courses (Introduction and Infection Prevention and Control) attracted more than 200,000 learners each in less than 2 months.

**Figure 8:**
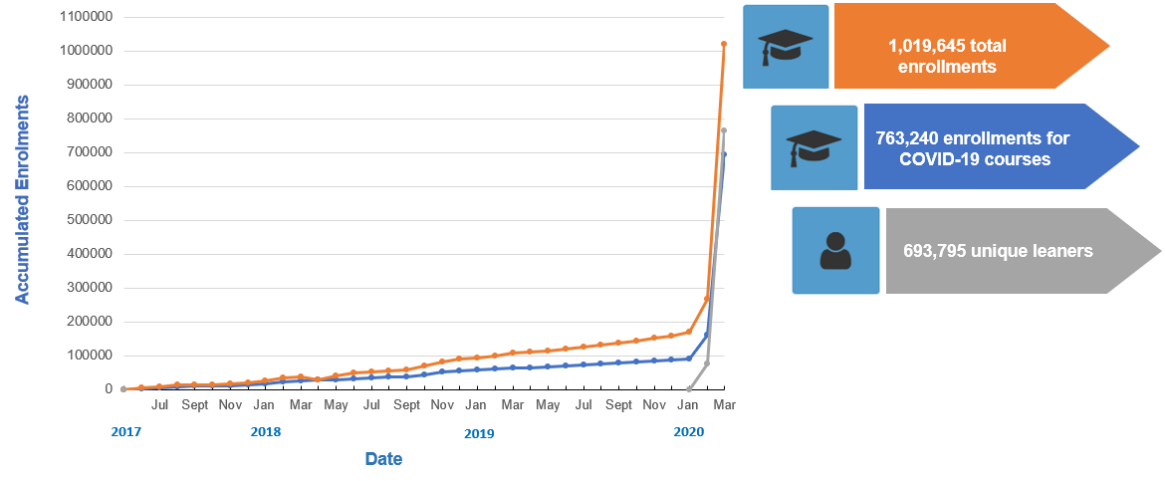
OpenWHO accumulated enrollments from June 30, 2017, to March 27, 2020. COVID-19: coronavirus disease.

Before COVID-19, there were on average some 100 course enrollments per day. During the first months of 2020, there were some 10,000-20,000 enrollments per day, with sharply increasing figures reaching up to 50,000 new learner registrations the week of the pandemic declaration (Figure 8). This testifies to the essence of OpenWHO offering health-related technical knowledge to frontline responders and the general public as an open and scalable solution for the fast distribution of lifesaving content in disease outbreaks and, in particular, during a pandemic.

**Figure 9:**
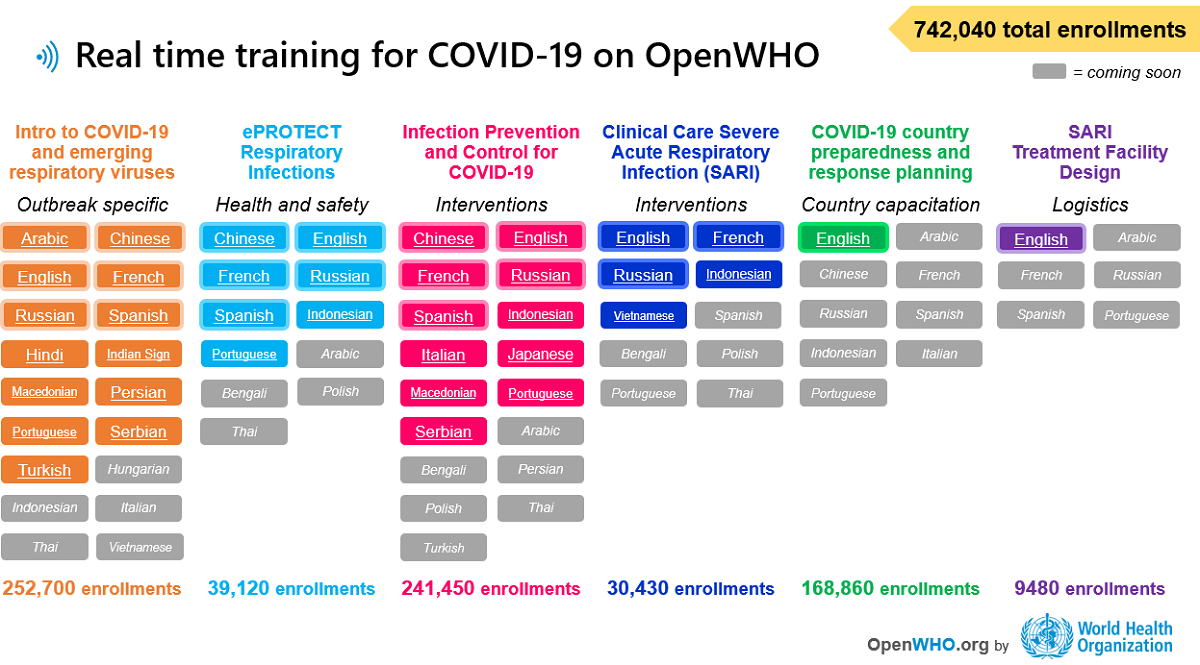
OpenWHO courses related to COVID-19 and language versions as of March 27, 2020. COVID-19: coronavirus disease.

OpenWHO has been working in full support of COVID-19 preparedness and response with the timely upload of learner resources, which was characterized by an accelerated process to make different language versions of the learning materials rapidly available. There was also an emphasis on quickly delivering the key available technical and operational information. Including the introductory COVID-19 course, a total of six courses were produced fully or partially in 40 different languages in the first quarter of 2020 (Figure 9).

During the early part of the coronavirus epidemic and pandemic, platform use shifted from health professionals and experts to largely non-health-related audiences. Between January 26 and February 25, 2020, OpenWHO expanded from some 80,000 existing unique users to 160,000, doubling the number of learners. From February 26 to March 25, 2020, the unique user numbers almost quadrupled to 600,000. Including the enrollments in the same courses hosted on PAHO’s VC platform, there were more than 840,000 enrollments in all of the COVID-19 courses.

**Figure 10:**
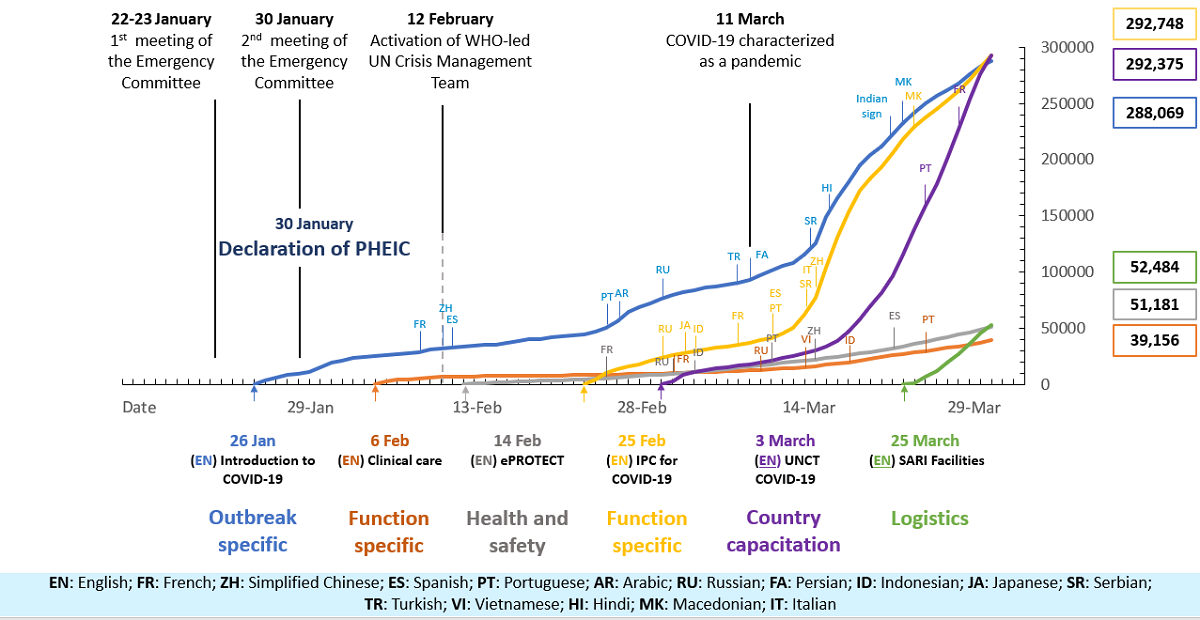
Timeline of all OpenWHO.org COVID-19 courses' launch and use. COVID-19: coronavirus disease; IPC: infection prevention and control; PHEIC: Public Health Emergency of International Concern; SARI: severe acute respiratory infection; UNCT: UN country teams; WHO: World Health Organization.

After the declaration of the pandemic on March 11, 2020, the number of unique learners on the platform nearly tripled in 2 weeks, from 235,250 users to 629,500 users as of March 25. The increase from March 11 to March 25, 2020, consisted of a total of 394,250 new learners in merely 14 days. This number is more than four times higher than the 90,700 total users on the platform from 2017 to 2019 (Figure 10).

## Discussion

### Principal Findings

The OpenWHO learning resources are usually initially published in English, as this language is shared by many native and nonnative speakers across the world and is the most commonly used working language of the UN system. Recent trends seen on the platform during the pandemic, notably the popularity of the Spanish versions of the COVID-19 resources surpassing that of the original English courses, stressed the importance of a multilingual learning platform that allows learners to access information in the language they are the most comfortable using. This has been a continuous effort since the platform’s inception, with courses even produced in local languages for localized emergencies. During the Ebola outbreak in the Democratic Republic of the Congo, for example, the Ebola knowledge resources for the responders course was published in Lingala and Swahili.

This multilingual approach is even more important in the middle of a pandemic, where OpenWHO is experiencing record-breaking enrollments from learners across the world who are seeking the latest WHO guidance to support the preparation and response to COVID-19. Indeed, in its first 2 months, the introductory course to COVID-19 comprised more than a quarter (n=241,749) of the accumulated total enrollments on a platform that has been running since June 2017. The analysis also indicated that substantial numbers of people were using the resource in languages other than the national languages of the countries in which they were located, suggesting that the platform provides a service that local governments may not be able to offer. With diaspora populations scattered across the world, the ability to access material in one’s native language irrespective of location is important.

The geographic analysis demonstrated that the enrollment surge for this emergency-related course mainly originated from Central and South America, with Ecuador, Mexico, Columbia, Chile, and the United States rounding out the top 5 countries, reflecting a new demographic that is attracted to the platform. Interestingly, the age distribution analysis for the Spanish course, with enrollments mainly concentrated in the Region of the Americas (n=98,554/103,207, 95.49%), revealed the popularity of the course with those 70 years or older compared with the other courses hosted on the OpenWHO platform. This is consistent with the at-risk group seeking reliable knowledge on the emerging respiratory disease. Prior to the pandemic, the top countries for OpenWHO’s most popular emergency courses were on the African continent, along with recurrent appearances by the United States and India, as these populous countries are big MOOC users.

This geographic shift is consistent with the nature of a pandemic. Rather than affecting specific parts of the world, as was the case for the 2014 Ebola outbreak, for example, the COVID-19 epidemic has accelerated into a pandemic reaching almost every country on the planet.

In contrast with the top emergency courses prior to the COVID-19 outbreak, the analysis also revealed that nearly 30% of users indicated that they were not affiliated with the student and professional health sectors (n=16,527/52,214, 31.65%). This shift reflects the impact a pandemic has on the audience profile, with the general public enrolling in the course to become informed on the novel coronavirus. It also suggests that OpenWHO can serve as one mechanism to help combat the “infodemic” of misinformation that has occurred during the COVID-19 outbreak [10]; research has found that many people turn to the internet for health information, including during crises, and the information they find can influence health behaviors [11-13].

OpenWHO hit the 1 million enrollments milestone on March 27, 2020. About three-quarters of the total enrollments were on the courses related to COVID-19. This reflects a massive increase in the popularity of OpenWHO and the critical role it is playing in supporting preparedness and response during this unprecedented pandemic. As the outbreak continues to evolve, new resources and language versions will be added to the platform to provide lifesaving knowledge to affected communities, and existing courses will be updated to best reflect the changing contexts.

### Limitations and Future Research

This analysis was limited to the 2-month period following the launch of the introductory COVID-19 course on OpenWHO. Data such as affiliation, gender, and age were only available for 32.43% (52,214/161,007) of the users who indicated their background. The geographical data was based on the users’ internet protocol addresses, which would not account for potential manipulation by virtual private networks or other factors. In addition, completion rates were measured by user visits to each of the course’s learning items, as quizzes were not yet implemented due to the evolving nature of the emergency guidance.

Future research should examine the use case of additional COVID-19 courses on the OpenWHO platform that are more targeted to specific audiences, such as courses designed for clinicians and public health professionals. Data should also be analyzed against the worldwide, regional, and country-level epidemic curves for COVID-19 to identify broader use trends, including how the use case evolved throughout the pandemic, as different regions were more or less affected.

### Conclusions

During health emergencies, lifesaving information must be packaged and delivered in the languages spoken by the target audiences to effectively transfer urgent knowledge [4,5]. Everyone has the right to access lifesaving knowledge, and OpenWHO is continuing to work with partners to make its resources available in as many languages as possible. The OpenWHO team has never before experienced such high levels of volunteerism for platform language production and has relied on crowdsourcing across the world to publish additional language versions. This has promoted the localization of materials into a variety of languages, helping people better protect themselves and fight the pandemic.

The OpenWHO platform offers courses on six distinct topics to support the COVID-19 response. These are products that transform the WHO guidance into learning packages that users can grasp and digest more easily. The courses have been translated and published fully or partially into 40 language versions during the first 2 months of the response, and OpenWHO has experienced an unprecedented increase in platform use. Amidst huge demand for reliable resources that offer the knowledge to understand and decipher the evolving situation, OpenWHO has served as one source of digitized information.

## Abbreviations

COVID-19: coronavirus disease
MOOC: massive open online course
PAHO: Pan-American Health Organization
WHO: World Health Organization
VC: virtual campus
